# Multiscale modelling of cage effects on the transport of effluents from open aquaculture systems

**DOI:** 10.1371/journal.pone.0228502

**Published:** 2020-03-23

**Authors:** Ole Jacob Broch, Pascal Klebert, Finn Are Michelsen, Morten Omholt Alver

**Affiliations:** 1 SINTEF Ocean, Trondheim, Norway; 2 Dpt. of engineering cybernetics, Norwegian University of Science and Technology, Trondheim, Norway; University of Waikato, NEW ZEALAND

## Abstract

Most Atlantic salmon mariculture operations use open sea cages for the grow out phase. The ultimate fate and effects of the effluents and the possibilities of disease transfer between fish farms are major concerns for farmers, governance and the general public alike. Numerical model systems applied to studying and managing effluents and disease transfer in mariculture must realistically resolve the hydrodynamics in the vicinity of the fish farms. In the present study, the effects of the aquaculture structures on the current patterns were introduced in the ocean model system SINMOD. The drag parameters for the ocean model were determined by comparing the simulation results from the ANSYS Fluent ^®^ software suite and SINMOD in an idealized channel setting with uniform currents. The model was run for a number of realistic scenarios in high horizontal resolution (∼30 m) with sea cages influencing the flow field. Comparisons between extensive current measurements and the simulation results showed that the model system reproduced the current local current field well. By running simulation scenarios with and without the effects of the sea cages on the flow field, it was possible to assess the importance of such effects for numerical dispersal models and aquaculture environment interactions simulations and hence for assessment of environmental impacts.

## Introduction

Numerical simulation models are standard and indispensable tools in studies and assessments of environmental effects and interactions of open water aquaculture systems [1, 2]. Some simulation tools are, directly or indirectly, integrated in management systems [3–6]. Common to all such simulation models is that they need to reasonably account for the hydrodynamics at and around the sites involved. While single point current measurements can be appropriate for rough local or near-field assessments, properly resolved hydrodynamics, that must necessarily be supplied by models, are needed for simulation of far-field dispersal in topographically complex and dynamic environments [7, 8].

Numerical challenges that arise at an appropriate spatial resolution are the effects of the aquaculture structures themselves on the hydrodynamics, how to handle such effects in a model and whether this is of any importance at all. This has been investigated for bays of extensive aquaculture installations using 2D models [9–11], indicating a potentially significant effect of the installations. 3D simulations have indicated a potential for significant increases in the erosion of the seabed sediments when including aquaculture installation effects due to acceleration of the currents underneath the farm [12] (though without explicitly simulating transport). Very detailed and high resolution modelling of the dispersal of fish farm nutrients has been performed for an idealized embayment [13], while the modification of currents by suspended canopies have been studied in [14]. Coupled biophysical models have been applied in detailed, high resolution studies of dispersal of nutrients from Atlantic salmon (*Salmo salar*) farms, but without considering the farm structures [15].

In the present study the effects of aquaculture installation structures on the hydrodynamics have been implemented for the nested 3D ocean model system SINMOD [7, 16]. The effects have been parametrized from Computational Fluid Dynamics (CFD) simulation results using ANSYS Fluent^®^, thus transferring information for a local fine-scale model to a high resolution ocean model. A with realistic bathymetry and topography for a Norwegian coastal region that is intensively farmed for Atlantic salmon has been established, allowing for dispersal simulations and studying the importance of including the farm structure effects on the hydrodynamics. In particular the aim of this study is to include likely effects on hydrodynamics while still being able to simulate important local features and far-field dispersal. While it can be difficult to determine precisely the impacts of the farm structures, including the fish biomass, on the hydrodynamics by current measurements alone [17], the effects are apparent from Eulerian tracers. The importance of including farm structure effects in near- and far-field dispersal simulations is discussed, as well as the implications for simulation based assessment and management of water connectivity and the potential for pathogen transfer between farms.

## Materials and methods

**Fig 1:**
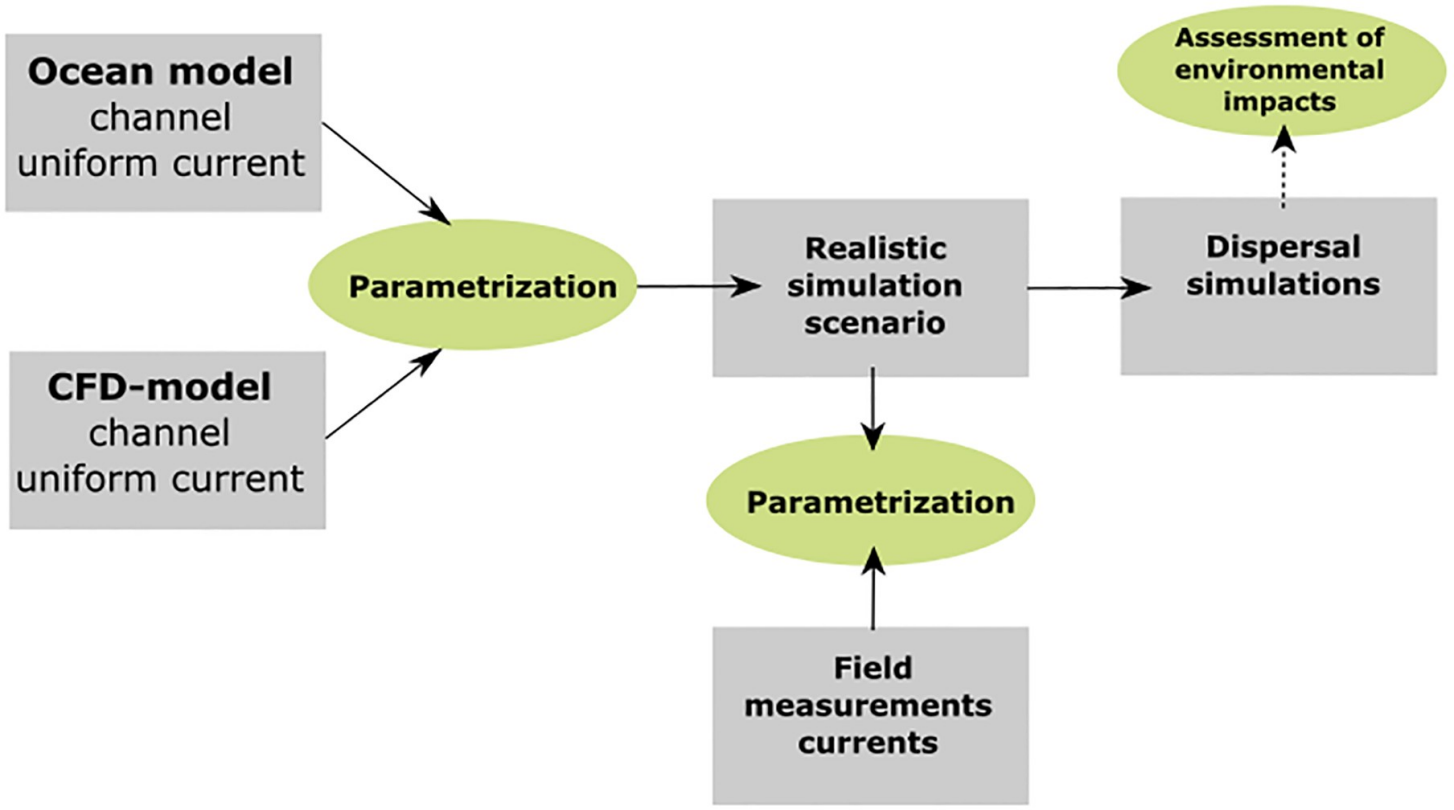
The concept of the study.

**Fig 2:**
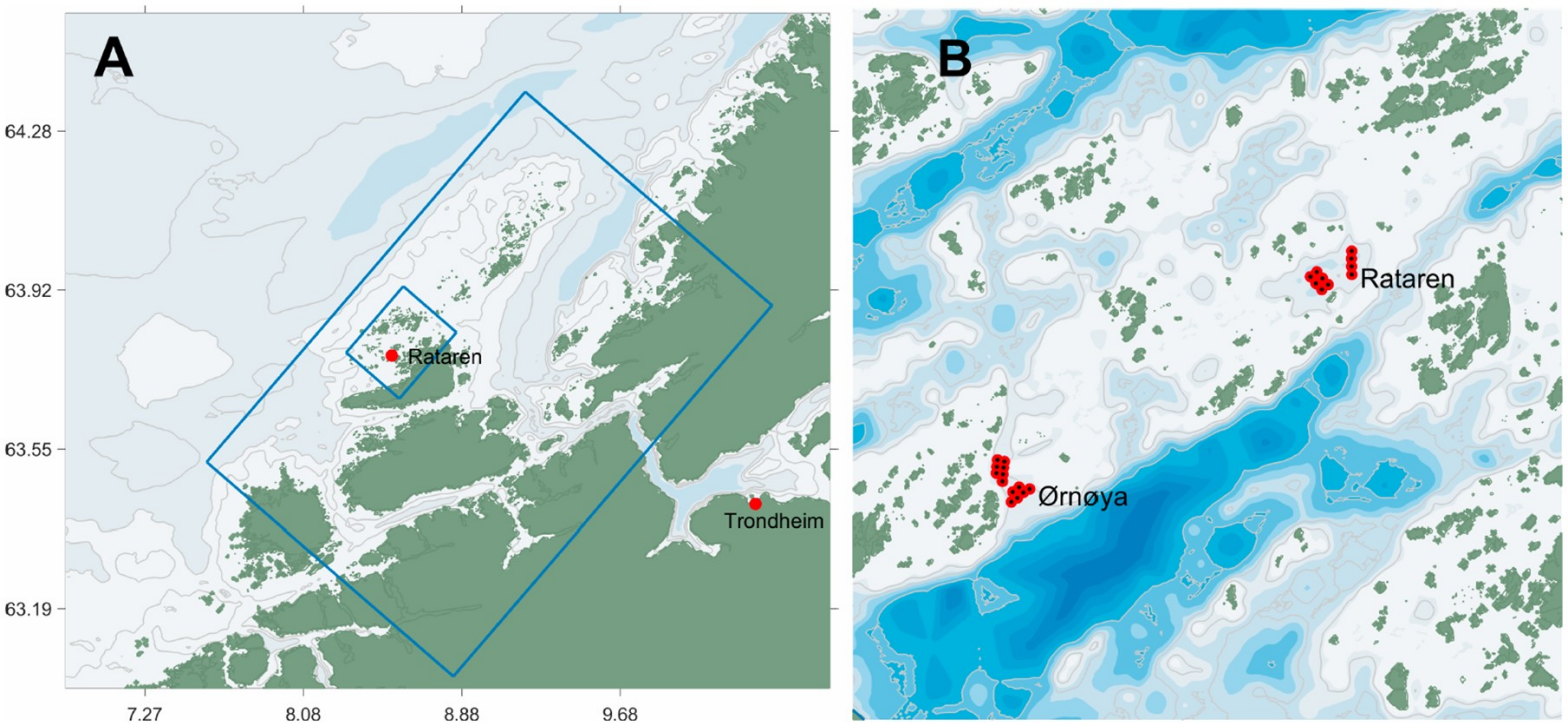
Study region and model domains. A: Model domains in 160 (large rectangular region) and 32 (small rectangular region) m horizontal resolution for the region around the aquaculture facilities studied. The city of Trondheim and the farm at Rataren (63.8°N; 8.5°E) are indicated. The region shown is a detail of the model domain of 800 m resolution used to generate boundary conditions for the 160 m model. B: *Detail* of the 32 m model domain with the farms Ørnøya and Rataren indicated. The distance between the centres of the farms is 4.3 km. The entire 32 m model domain represents a region of approx. 22 × 18 km.

A concept diagram for this study is shown in Fig 1. A drag parameter, representing the effect of porous structures in the water (e.g. fish cages), was introduced in the ocean model. The numerical value of the drag parameter was estimated by comparing the ocean model results for several values with high resolution CFD results for a similar idealized model setup, and selecting the drag parameter value giving the least difference in the simulation results of the two models. An ocean model scenario was established, including observed data on bottom topography, land contours, atmospheric forcing and freshwater runoff, as well as boundary conditions from larger model domains. The results from these simulations were compared with measurements from six stations around the fish farm at Rataren, Central Norway (Fig 2) for two two-month periods. The simulation model was then used to study changes to passive tracer concentration fields resulting from including fish farm effects.

### Ocean model SINMOD and the implementation of the effects of porous structures

The hydrodynamic calculations in SINMOD are based on the primitive Navier-Stokes equations. The horizontal acceleration in the *x*-direction is given by

$$\frac{\partial u}{\partial t}=fv-u\frac{\partial u}{\partial x}-v\frac{\partial u}{\partial y}-w\frac{\partial u}{\partial z}-\frac{1}{\rho }\frac{\partial p}{\partial x}+\nabla \left(A_{\text{h}}\nabla u\right)+\frac{\partial }{\partial z}A_{\text{v}}\frac{\partial v}{\partial z}+\sigma_{x}.$$ (1)

The equation for acceleration in the *y*-direction is similar [16]. Here, *u* and *v* denote the velocities in the *x* and *y* directions, respectively, while the vertical velocity *w* is calculated from the continuity equation for incompressible fluids. The Coriolis parameter is denoted by *f*. Pressure, *p*, is calculated from the hydrostatic equation. Calculation of the horizontal turbulent diffusion of momentum (*A*_h_) is based on Smagorinsky [16, 18]. The vertical mixing coefficient (*A*_v_) is based on a Richardson scheme [19]. SINMOD uses *z* layers, and is solved by a finite difference scheme on an Arakawa C-grid. See [16, 19–21] for the basic details on the model system.

The final term in (1), *σ*_*x*_, handles surface and bottom stress in addition to drag from other objects due to the current speed in the *x*-direction (*u*). In a discretized model setup with a maximum of *K* > 2 depth layers of thickness Δ*z*_*k*_ (1 ≤ *k* ≤ *K*) we let:

$$\sigma_{x}\left(k\right)=\frac{1}{\Delta z_{k}}\{\begin{array}{cc} \tau_{\text{surf}}-\tau_{\text{struct}}, & k=1\\ -\tau_{\text{struct}}, & 1<k<K\\ -\tau_{\text{bot}}-\tau_{\text{struct}}, & k=K. \end{array}$$ (2)

Here, *τ*_surf_ and *τ*_bot_ denote the surface (wind) and bottom stress, respectively [20], while the effects of structures are included via

$$\tau_{\text{struct}}=\delta_{\text{struct}}u_{k}\sqrt{u_{k}^{2}+v_{k}^{2}},$$ (3)

where *u*_*k*_, *v*_*k*_ are the components of the current velocity in depth layer *k* and *δ*_struct_ is the drag parameter for the object(s) in the model grid cell. The construction for the *y*-direction is analogous, using *v*_*k*_ in place of *u*_*k*_.

The presence of objects alters the volume for water flow (blockage effect). This is accounted for by a “porosity” parameter, 0 < *θ* ≤ 1, for the fraction of the model grid cell *not* occupied by any solid object. Thus, *θ* = 1 indicates that there is no object or blockage effect present. The original (without any obstruction present) vertical thickness *h*_*k*_ of a model grid cell is modified according to

$${\tilde{h}}_{k}=\theta_{k}h_{k}.$$ (4)

**Fig 3:**
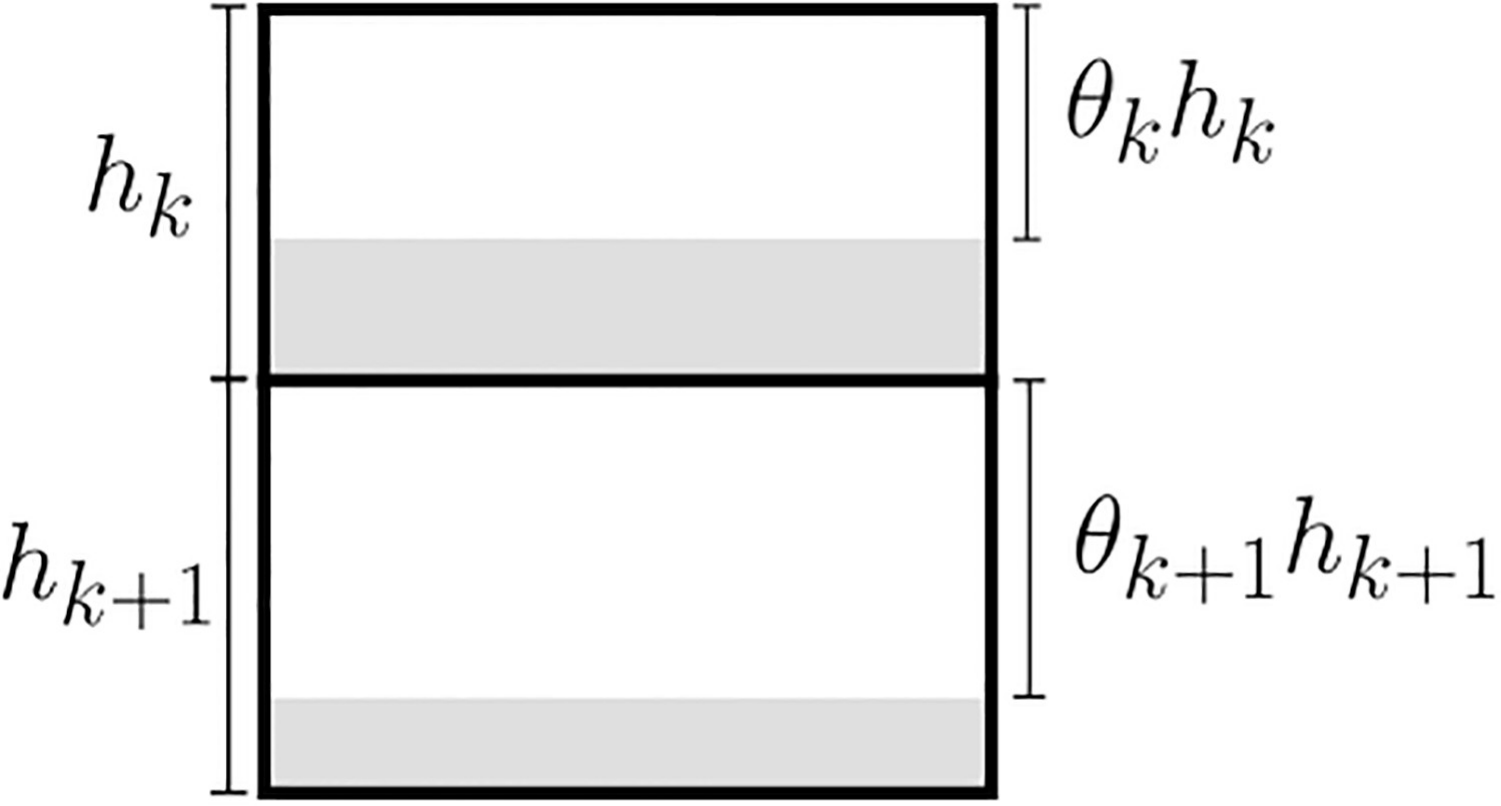
The porosity parameter. Illustration of the porosity parameter *θ* for two adjacent depth layers in a specific horizontal position. The thickness of depth layer *k* is denoted by *h*_*k*_. The gray regions indicate the total volume occupied by one or more objects in the cell. The unoccupied volume fraction is denoted by *θ*_*k*_, *θ*_*k*+1_. This fraction is used to modify the available water volume used in the calculations.

See Fig 3. A similar approach for a depth integrated model is used in [11].

The numerical values of *θ* and *δ*_struct_ must necessarily represent average values over entire model grid cells. Thus, these parameters depend not only on the type and size of the objects that they represent, but also on the model grid resolution. Ocean models typically have horizontal resolutions ranging from ∼ 10^1^ to ∼ 10^3^m.

### CFD model

CFD models basically solve the same equations as ocean model systems, but usually on different spatial and temporal scales. Typically, the grid resolution is of the order of magnitude ∼ 10^−1^ to ∼ 10^1^m. In the present case, the computational domain described in the next subsection has been used for these simulations. The ANSYS Fluent ^®^ (16.4) software package was used. A grid generator (Workbench) has been used to generate unstructured (around and inside the cage) and structured grids (in the rest of the 3D domain). Pressure jump boundary conditions [22] have been applied to the netting by using a porous model acting like a membrane and adding resistance to the flow. A k-*ϵ* turbulence model [23] has been used to perform these flow simulations. A velocity reduction of 20% through each net is simulated, resulting in roughly a 40% current flow reduction through the entire cage as measured by [24].

### Parametrization of the ocean model

**Fig 4:**
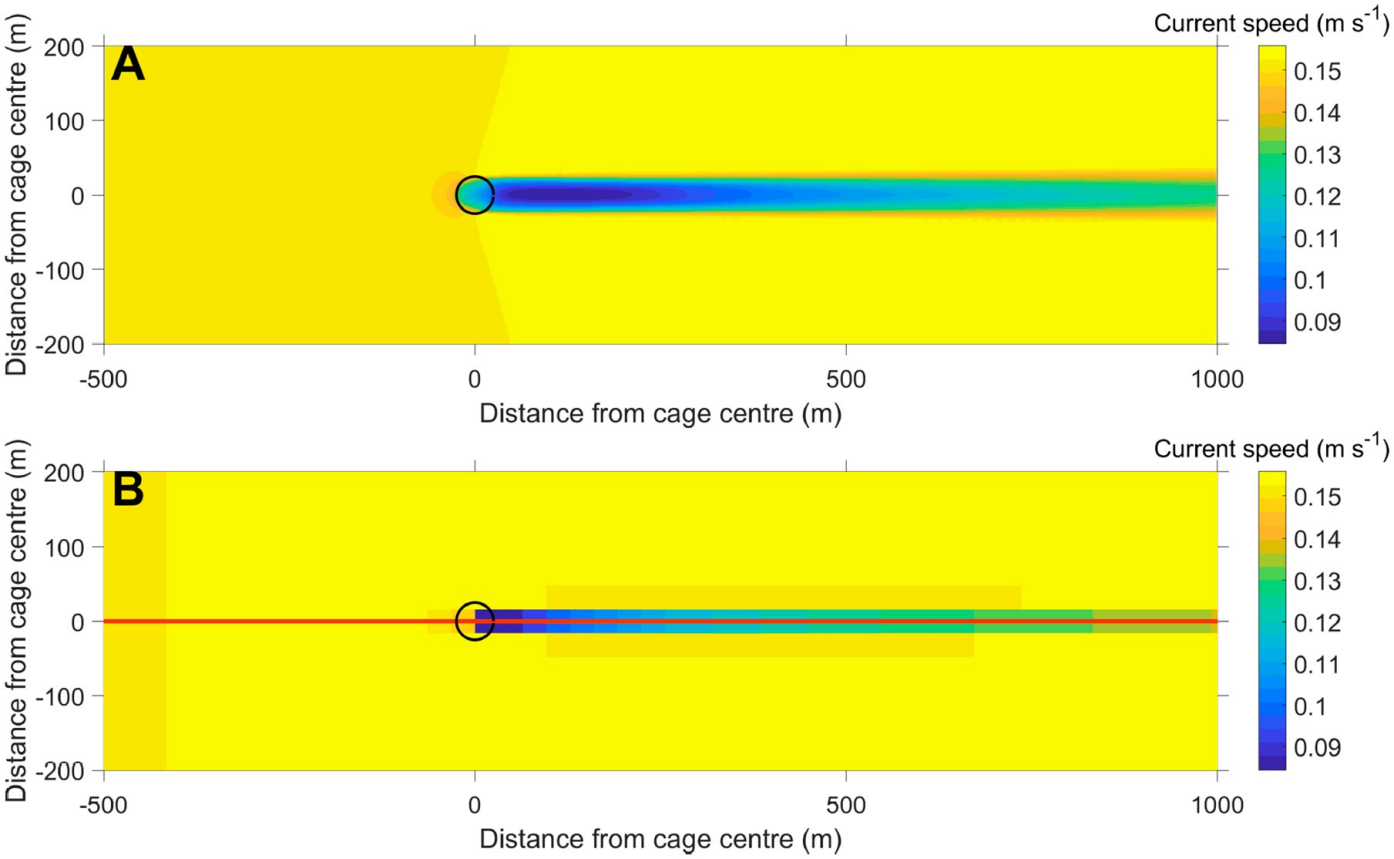
Wakes of salmon net pens. Comparison of the 10 m depth horizontal cross sections of the wakes of a circular salmon net pen (of diameter 50 m, 25 m deep, indicated by black circles) simulated by FLUENT ^®^ (A) and SINMOD (B). The red line in B indicates the cross section used in the comparison of the CFD and ocean models and the parametrization of the drag coefficient (Fig 6, Eq (5)).

Idealized model domain setups were established for ANSYS Fluent ^®^ and SINMOD (Fig 4). The CFD domain consisted of a channel 55 m deep, 960 m wide and 1500 m long. A tetrahedral mesh of resolution ranging from 0.1 m close to and around the cage (in order to capture local flow details) to 8 m further away (in order to reduce the total number of cells) was used. The cage design was similar to the one used at the actual fish farm at Rataren (Fig 2). The SINMOD (ocean model) domain had a horizontal resolution of 32 m, with uniformly spaced vertical layers of 1 m thickness and 133 × 30 horizontal grid cells. The Coriolis parameter (1) was set to *f* = 0 for these idealized scenarios. For the SINMOD simulations initial constant salinity of *S* = 34.9PSU and temperature of *T* = 10.9°C were specified, appropriate for the part of the Norwegian coastal waters that was of interest here [25].

The models were both run with constant entry speeds of *u*_0_ = 0.05, 0.1, 0.15, 0.20 and 0.40 ms^-1^ from the left. SINMOD was further run with different numerical values of the drag parameter: *δ* = 0, 0.01, …, 0.15 for each of these six current speeds.

The difference in output between the CFD simulations and the ocean model was quantified by calculating the RMS-difference in normalized simulated current speeds along a lateral transect through the farm (in the *x*-direction; Fig 4). Explicitly, denote by *s*_*i*,OM_(*u*_0_, *δ*) the current speed in grid cell number *i* along the lateral transect of the SINMOD domain for the scenario with entry speed *u*_0_ and drag coefficient *δ*, and by ${\bar{s}}_{i,\text{CFD}}\left(u_{0},\delta \right)$ the average current speed of the CFD model in the corresponding regions. The average is used for the latter model due to the much higher resolution of the CFD than the ocean model, so that each ocean model grid cell contains several CFD model cells (Fig 4). We calculate the normalized RMS-difference for each choice of numerical value of the drag parameter *δ* as follows.

$$\text{RMSD}\left(\delta \right)=\sum_{u_{0}}\sqrt{\frac{1}{n}\sum_{i=1}^{n}{\left(\frac{s_{i,\text{OM}}\left(u_{0},\delta \right)-{\bar{s}}_{i,\text{CFD}}\left(u_{0},\delta \right)}{u_{0}}\right)}^{2}}.$$ (5)

In the present case *n* = 47, i.e. there were 47 SINMOD grid cells along the lateral transect. The parameter *δ*_struct_ was then chosen as the parameter value of *δ* minimizing RMSD such that

$$\text{RMSD}\left(\delta_{\text{struct}}\right)=\underset{\delta }{\text{min}}\;\text{RMSD}\left(\delta \right).$$ (6)

This provides a parameter independent of the current speed.

### Ocean model setup for the Rataren region

SINMOD has previously been established for the region around the fish farm at Rataren [7]. The model domain covers a region of approx 22 × 18 km in a horizontal resolution of 32 m, and uses depth layers of 0.5 –2 m thickness from the surface down to 25 m depth and further layers of 5 –25 m thickness from 25 to 250 m depth.

Freshwater discharges from rivers and land were applied using data from simulations by the Norwegian Water Resources and Energy Directorate (www.nve.no) with a version of the HBV-model [26].

The model was nested in a four step procedure, nesting from a 20,000 m resolution model covering the northern North Atlantic and the Arctic seas down to 32 m (e.g. [7] and Fig 2).

For the high resolution (160 and 32 m horizontal resolution) ocean model domains, atmospheric forcing was taken from MEPS (www.met.no), while the coarser ocean model domains (800, 4,000, and 20,000 m horizontal resolution) were forced by ERA Interim data from ECMWF [27].

The 20,000 m model was forced with tidal components M_2_, S_2_, N_2_, K_2_, K_1_, O_1_, P_1_, Q_1_, Mf, Mm, and SSa at the open boundaries, with data on global ocean tides imported from the TPXO 7.2-model (http://volkov.oce.orst.edu/tides/TPXO7.2.html).

Previous comparisons between simulation results from SINMOD and current profiler measurements at the Rataren fish farm have been made in [7] and [17].

### Current measurements

**Fig 5:**
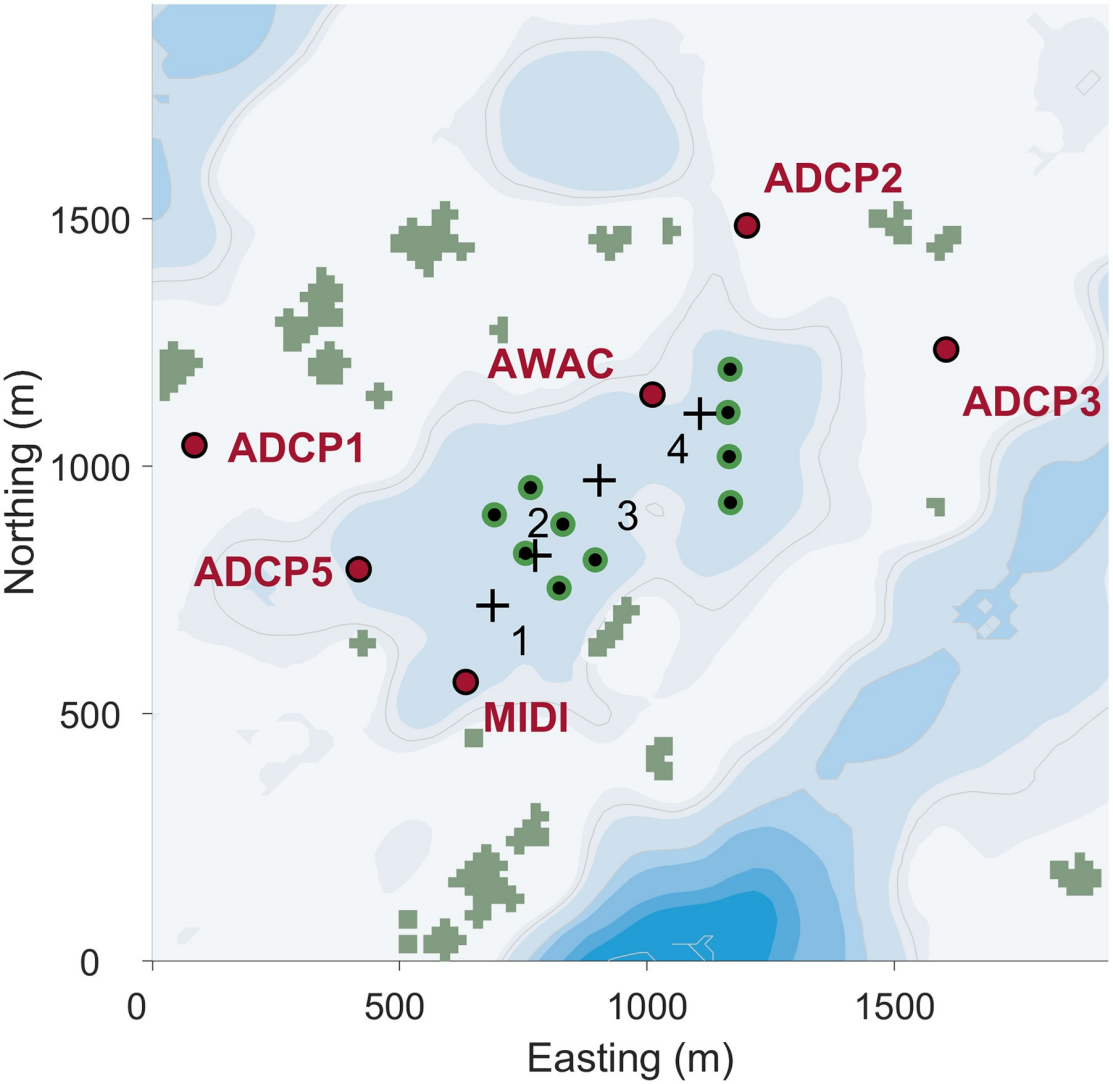
Layout of the farm at Rataren. The farm is located at 63.8°N; 8.5°E. The position of the cages (green and black dots) and the profilers (red dots) are indicated. The background colors indicate bottom depth (the darker, the deeper), and the thin gray curves are 25, 50 and 100 m isobaths. Land points are olive colored.

Water currents were measured at six points around the farm at Rataren (Fig 5) for two periods (Aug-Oct 2015; May-June 2016) using ADCPs and MIDI profilers. The sampling intervals for the ADCPs and MIDI buoy were 300 and 3600 s, respectively [17].

### Transport and dispersal simulations

Eulerian passive tracers were used to investigate the potential cage structure effects on dispersal of dissolved matter from the farms. A constant release of 1 unit PT per net pen per hour was used. The tracer was released homogeneously from the surface to the bottom of each net pen, assumed to be 25 m deep. Simulations were run with and without taking into account the effects of the cage structures on the water currents, for both of the fish farms Ørnøya and Rataren (Fig 2) and for two one-month periods: August-September (2015) and May-June (2016). None of the simulation scenarios were based on concrete production cycles at any of the two farms. Thus, the relative release rates of PT from the different cages may potentially differ from that of any actual production cycles.

Though passive, i.e. not equipped with any half-life or degradation rates, the tracers can be interpreted as dissolved nutrients like ammonium or phosphorous, pharmaceuticals, and as such will provide an upper estimate for concentrations and potential for water contact.

## Results

### Parametrization of the ocean model

The results from the idealized Fluent^®^ and SINMOD simulations for *u*_0_ = 0.15 ms^-1^ are shown in Fig 4, with *δ* = 4 × 10^−5^ for the SINMOD results. The CFD simulations indicate a reduction of the current speed at 10 m depth down stream from the farm with a fraction of 0.44, while the corresponding fraction in the SINMOD simulations was 0.46.

**Fig 6:**
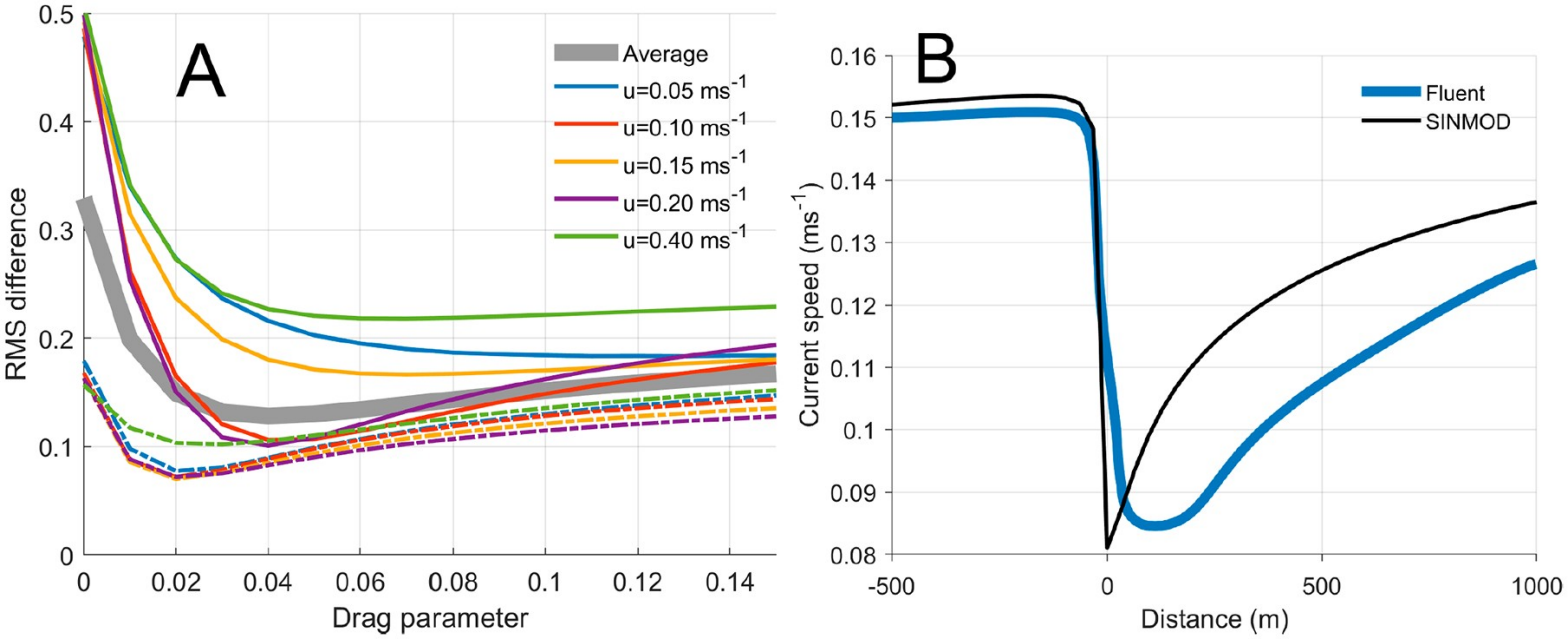
Estimating the drag parameter. A: The RMS difference in current speeds calculated by Eq (5) along lateral horizontal cross sections (Fig 4) at 10 (solid curves) and 20 (dashed curves) m depth between the SINMOD and FLUENT ^®^ simulations. B: current speeds along the lateral transect in Fig 4 at 10 m depth, from FLUENT ^®^ (blue) and SINMOD (black). Distances are relative to the center of the cage (Fig 4).

The thick, gray curve in Fig 6, A represents the compound, normalized RMSD calculated from Eq (5) as a function of *δ*. Corresponding curves for each of the entry current speeds *u*_0_ and each depth (10, 20 m) are also shown. The minimization by (6) yields

$$\delta_{\text{struct}}=0.04.$$ (7)

This can also be determined by inspection of Fig 6A. This numerical value of the parameter was used in the dispersal simulations described below in the “Dispersal of Eulerian passive tracers” below and in the comparison between the simulated current speeds along the lateral transect (Fig 4) in Fig 6B, by SINMOD and Fluent.

In a 32 by 32 m model grid cell, a fish net pen structure accounts for a small fraction of the volume only. The volume of a net pen of 50 m diameter, 25 m deep, is approx. 36,000 m^3^. Assuming a biomass of 1000 t, accounting for approximately 1000 m^3^, we thus have a potential blocking effect of up to 3%. Thus we set

θ=0.97. (8)

This is the value used in the hydrodynamic and passive tracer dispersal simulations. The model is not sensitive to changes in *θ* over the range (e.g. 0.9 < *θ* ≤ 1) that is relevant here.

### Currents and comparison of simulation results with current profiler measurements

**Fig 7:**
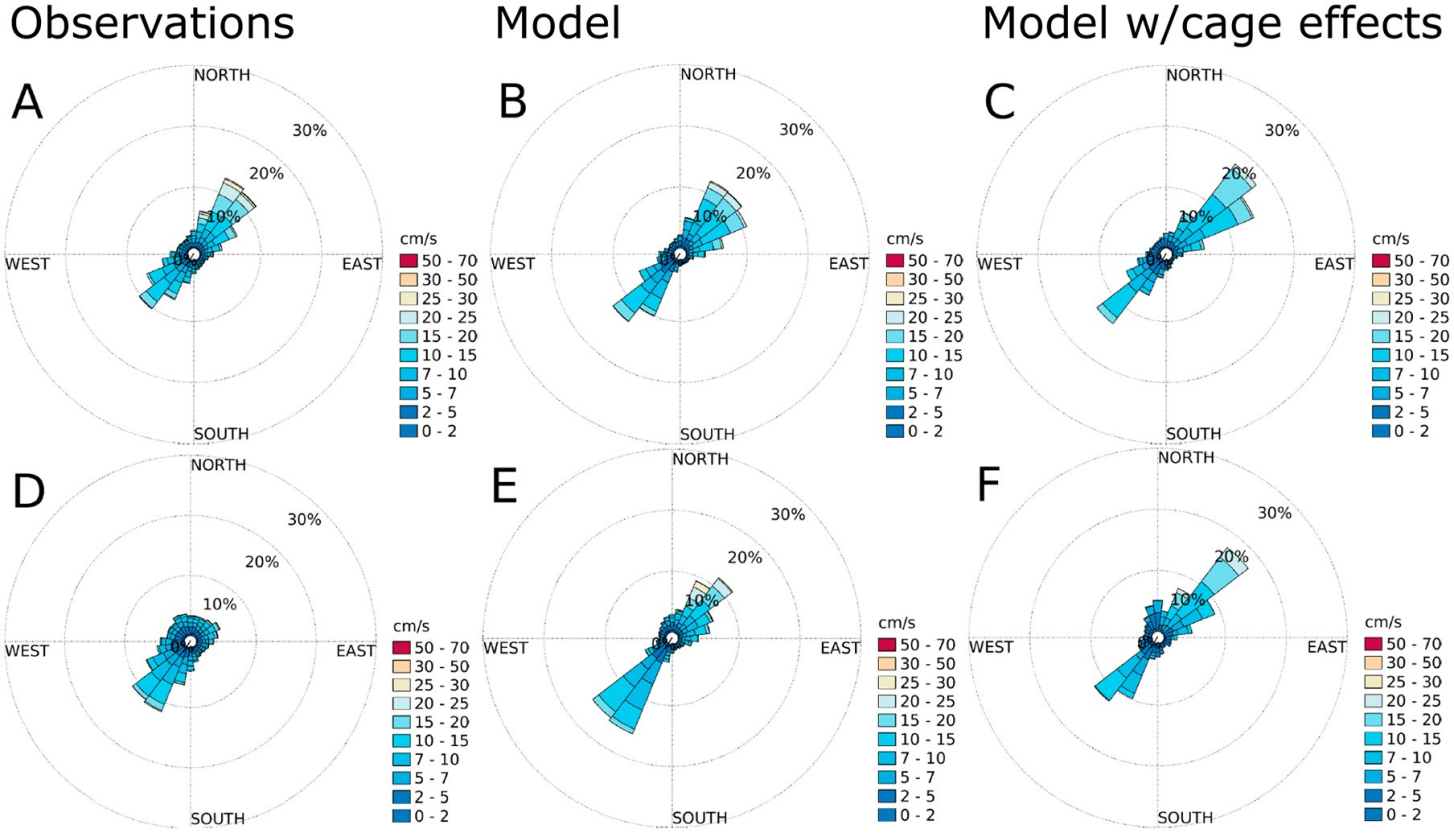
Current roses for ADCP5 (Fig 5) at 10 m depth. A: observations for August-October, 2015. B: simulation results without cage effects for August-October, 2015. C: simulation results with cage effects for August-October, 2015. D: observations for May-June, 2016. E: simulation results without cage effects for May-June, 2016. F: simulation results with cage effects for May-June, 2016. The colors indicate current speed. Each sector spans 15°.

**Fig 8:**
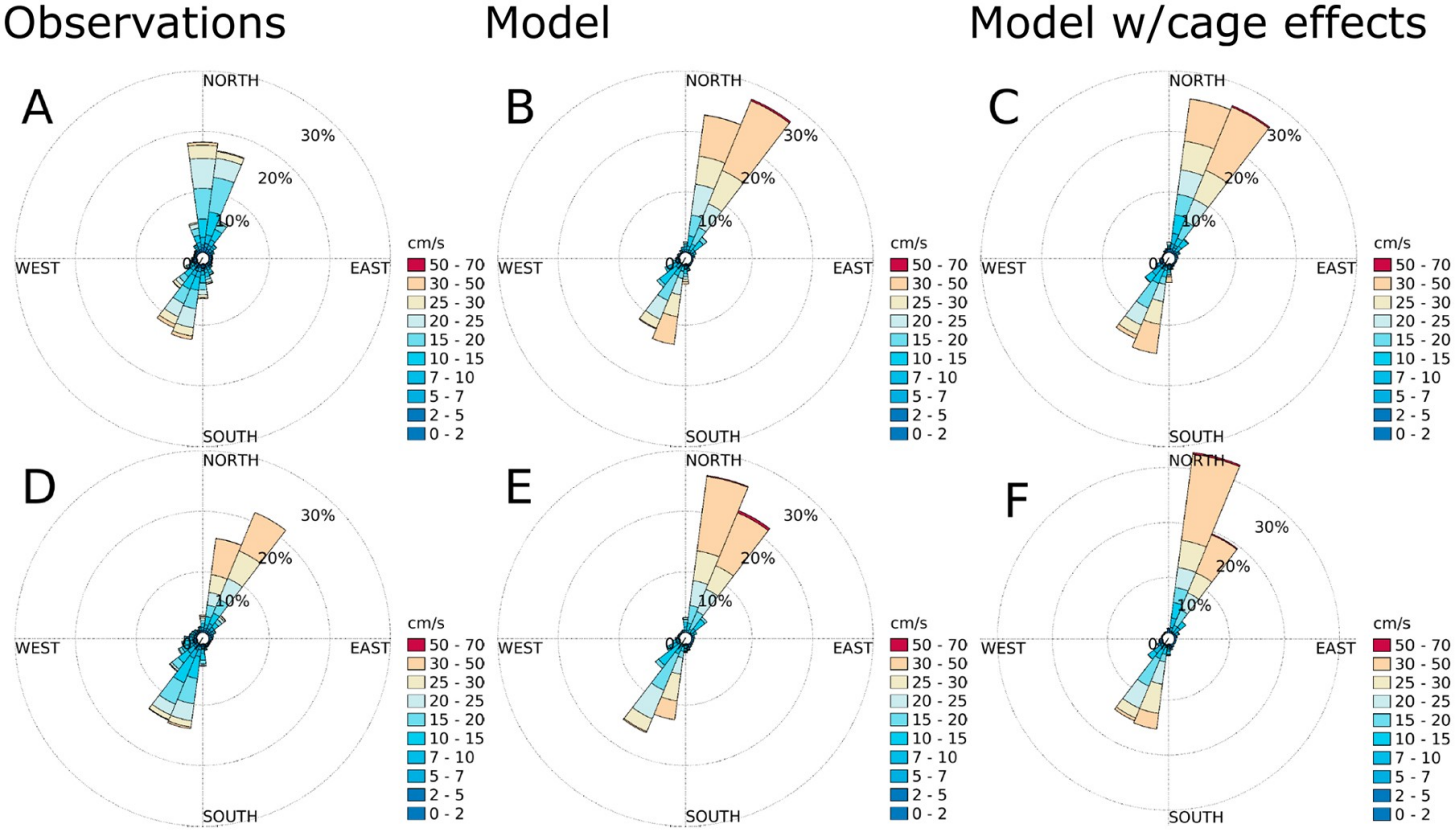
Current roses for ADCP2 (Fig 5) at 10 m depth. A: observations for August-October, 2015. B: simulation results without cage effects for August-October, 2015. C: simulation results with cage effects for August-October, 2015. D: observations for May-June, 2016. E: simulation results without cage effects for May-June, 2016. F: simulation results with cage effects for May-June, 2016. The colors indicate current speed. Each sector spans 15°.

**Fig 9:**
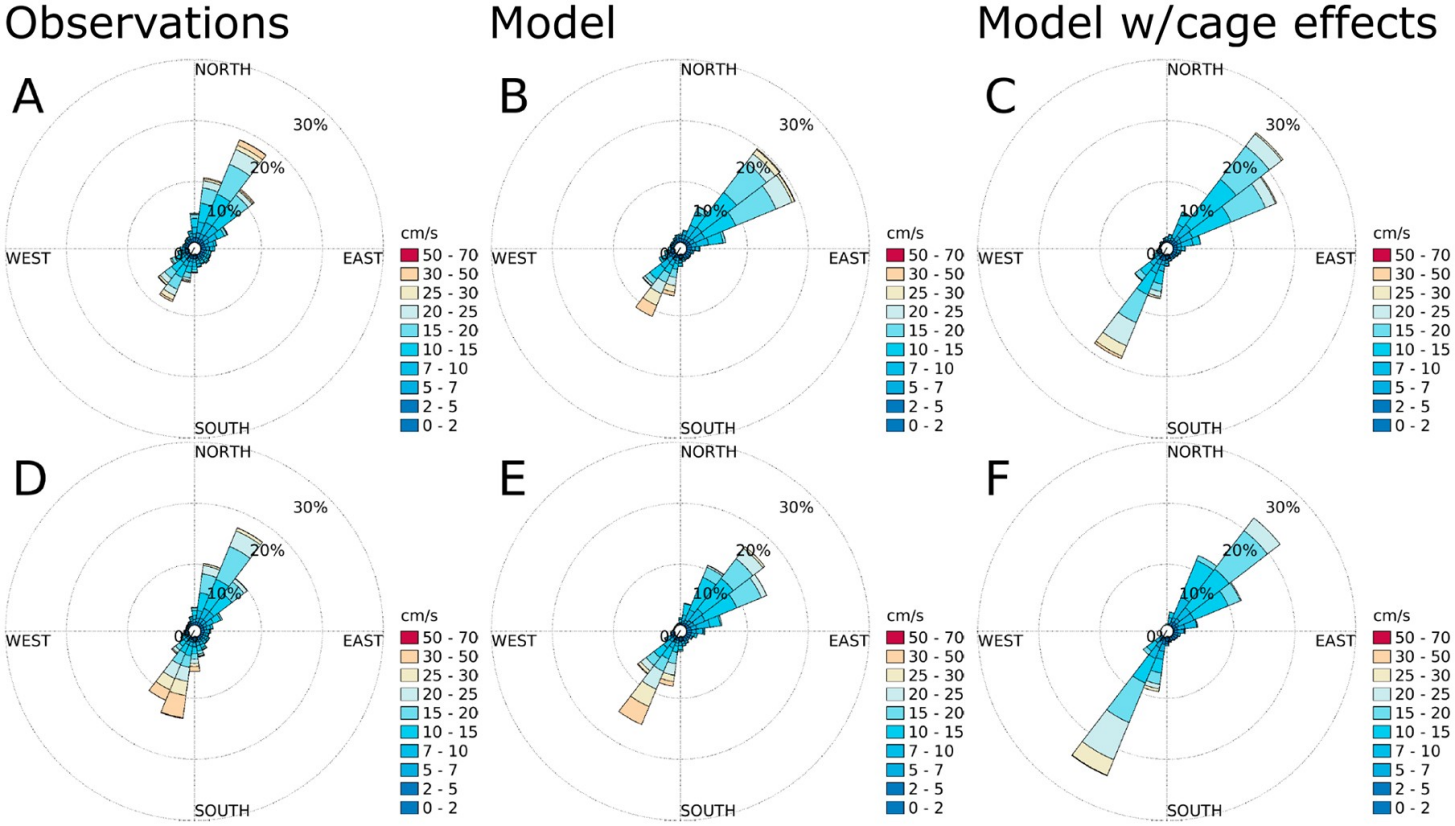
Current roses for the AWAC (Fig 5) at 10 m depth. A: observations for August-October, 2015. B: simulation results without cage effects for August-October, 2015. C: simulation results with cage effects for August-October, 2015. D: observations for May-June, 2016. E: simulation results without cage effects for May-June, 2016. F: simulation results with cage effects for May-June, 2016. The colors indicate current speed. Each sector spans 15°.

**Fig 10:**
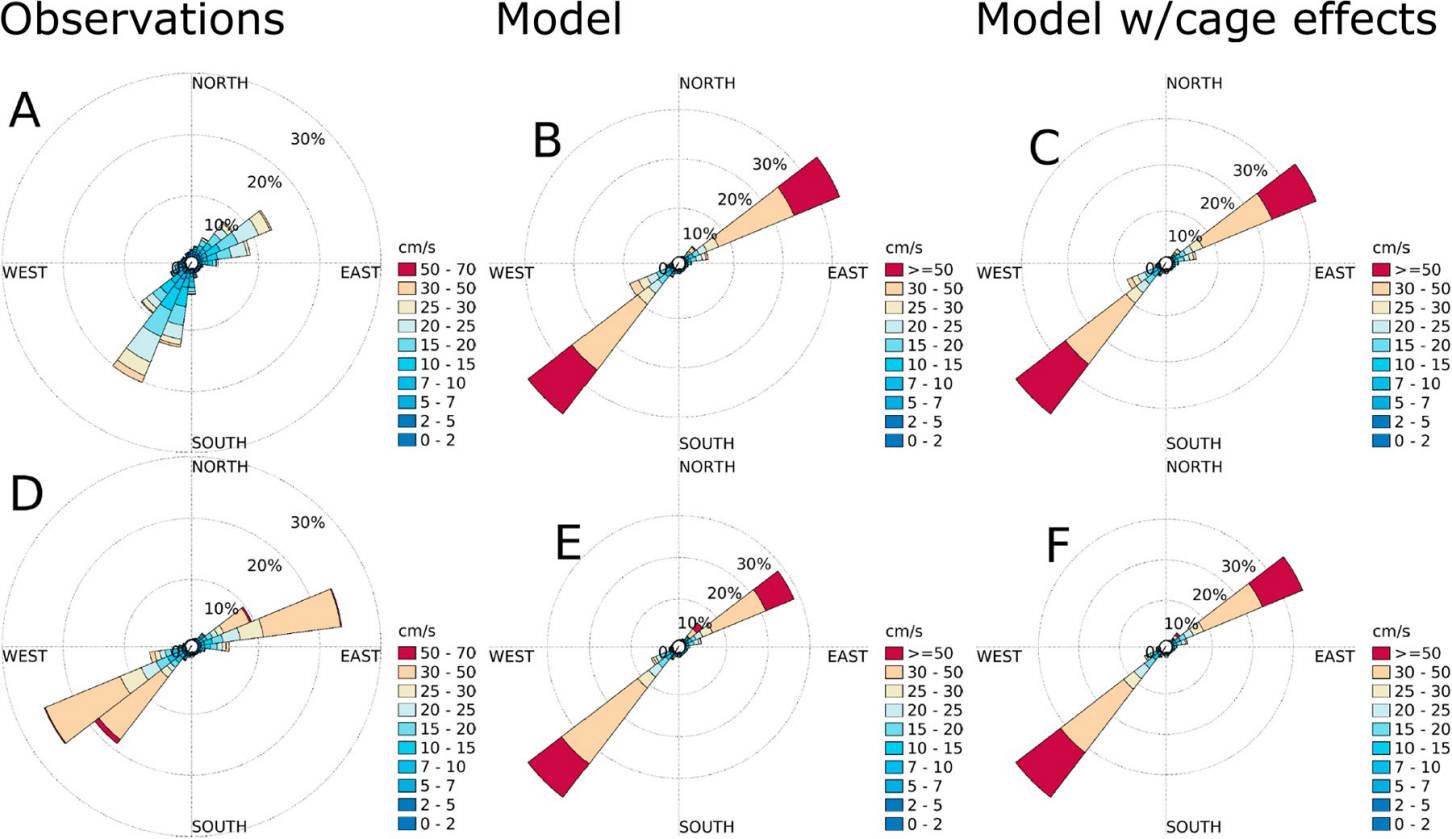
Current roses for ADCP3 (Fig 5) at 10 m depth. A: observations for August-October, 2015. B: simulation results without cage effects for August-October, 2015. C: simulation results with cage effects for August-October, 2015. D: observations for May-June, 2016. E: simulation results without cage effects for May-June, 2016. F: simulation results with cage effects for May-June, 2016. The colors indicate current speed. Each sector spans 15°.

According to the current measurements, the main flow direction was SW-NE at all stations (Figs 7–10), except at ADCP2 (Fig 8) in August-September 2015 with a stronger tendency for currents in the N direction and at ADCP3 (Fig 10) in May-June 2016 with a flow more in the Eastern direction. The simulation results matched the observations in terms of current direction at most of the stations, also in terms of the spread of the directions. An exception to this was ADCP3, with noticeably less spread in August 2015, and also higher current speeds. There was less spread in direction at ADCP3 in May, but with less difference in current speed between the observations and the simulation results than in the August results.

At ADCP1 (results not shown) the observations and simulations had similar current speeds, but the simulation results were a lot less spread out in direction than the observations. The site is very shallow and fairly close to land, and is a position where it is difficult to reproduce the situation in a model. There may also have been more noise in the observations at this sampling stations than at the others due to the shallow bottom depth.

The observations and simulation results were in agreement also at the MIDI buoy (not shown, see [17]).

An analysis of the current observations confirmed that the tide in the region is semi-diurnal with M2 the dominating component [28].

The effects of the fish cages was not very strong at either of the sampling stations (Figs 7–10), and there was no clear trend in terms of statistical significance. In some cases there was a significant change in the distribution of current speeds by including current reduction, in others not, when using the mean currents speed as the statistic. There was a tendency for less spread in the current directions when including the effects of the fish cages.

**Fig 11:**
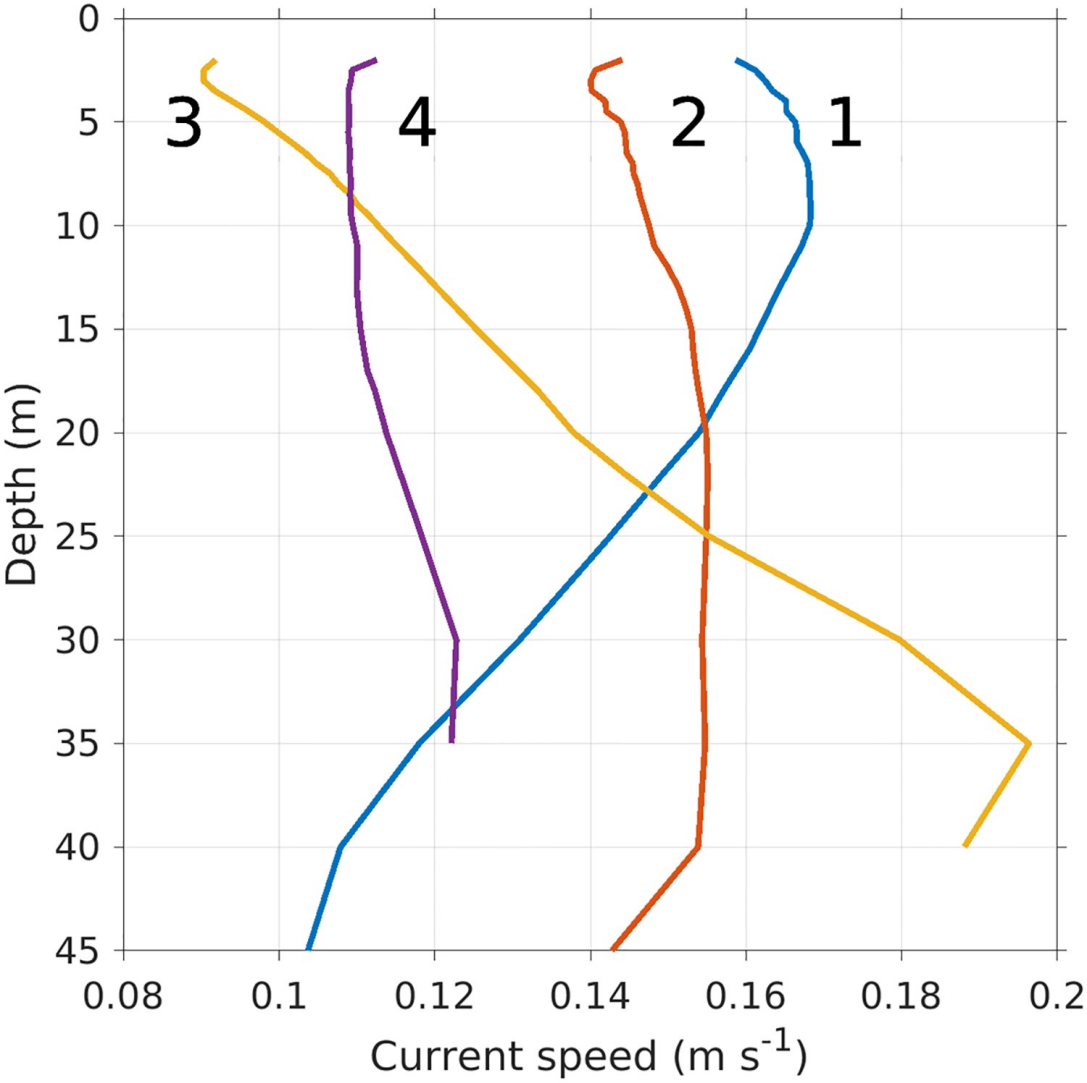
Depth profiles of simulated current speeds at the four “+”s numbered 1 to 4 in Fig 5. The profiles were averaged over a tidal cycle in August, 2015, when the current direction was roughly in the direction *from* +1 *to* +4. The large numerals at the top correspond to those in Fig 5.

During periods of more or less unidirectional currents through the farm (Figs 5 and 11), the effects of the cage arrays were apparent from the simulations results close by the farm. There was a reduction in the surface current speed and an increase in current speeds below the farm structure down stream of the farm (in the direction from +1 to +4 in Fig 5). Further down stream (from the scenario without to the scenario with cage effects (Fig 11)), this effect disappeared.

### Dispersal of Eulerian passive tracers

**Fig 12:**
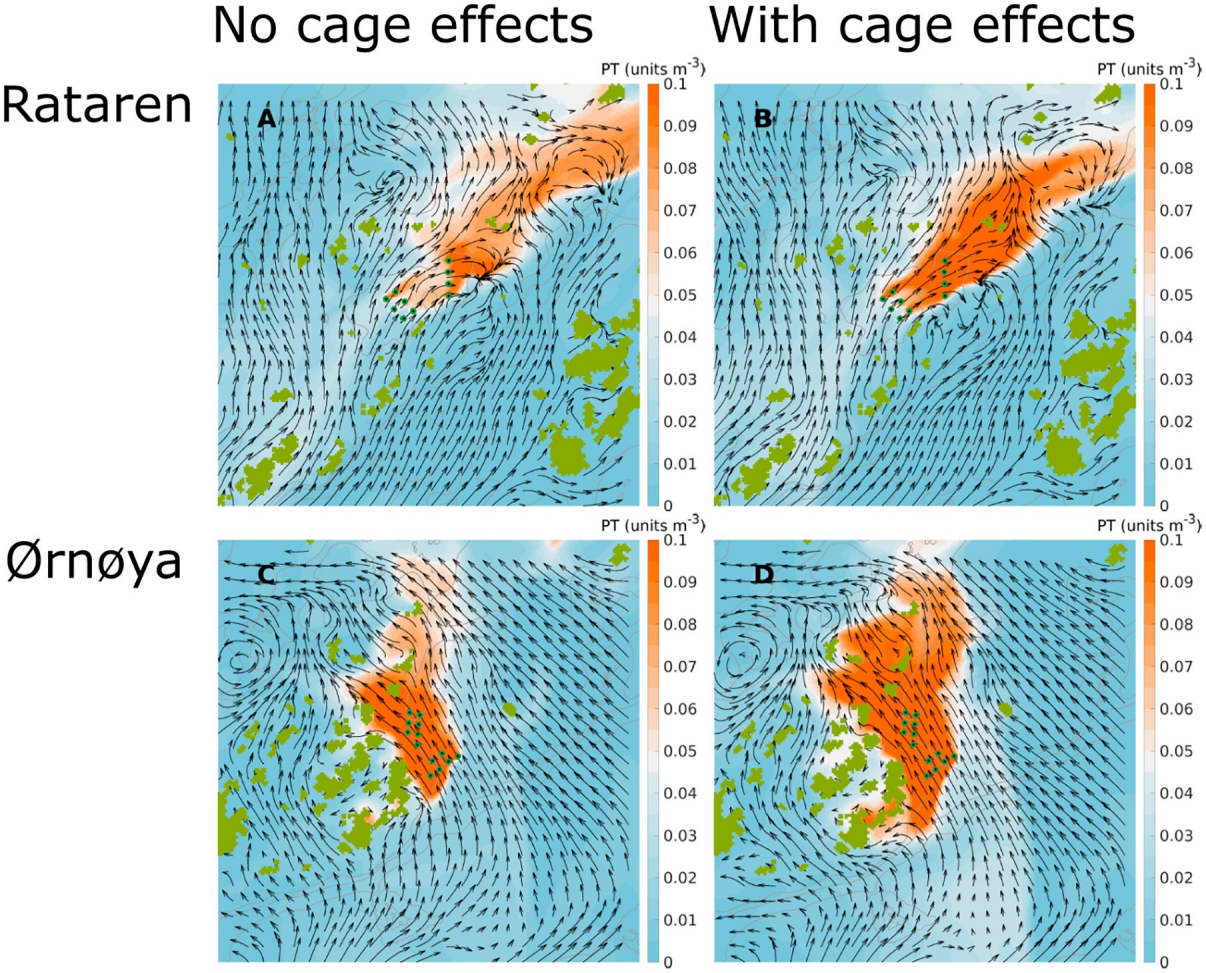
Simulated dispersal of passive tracers. Snap shots (instantaneous images, medio August 2015) of passive tracers released from the Rataren (A, B) and Ørnøya (C, D) farms with (B, D) and without (A, C) including the effects of the cages. The results are depth integrated (0 –10 m) passive tracer concentrations. The gray curves in the background are 30, 50 and 100 m isobaths. The curved arrows indicate the water current field. The release points of the passive tracer are indicated by green-black dots. The distance between them are approx. 100 m.

**Fig 13:**
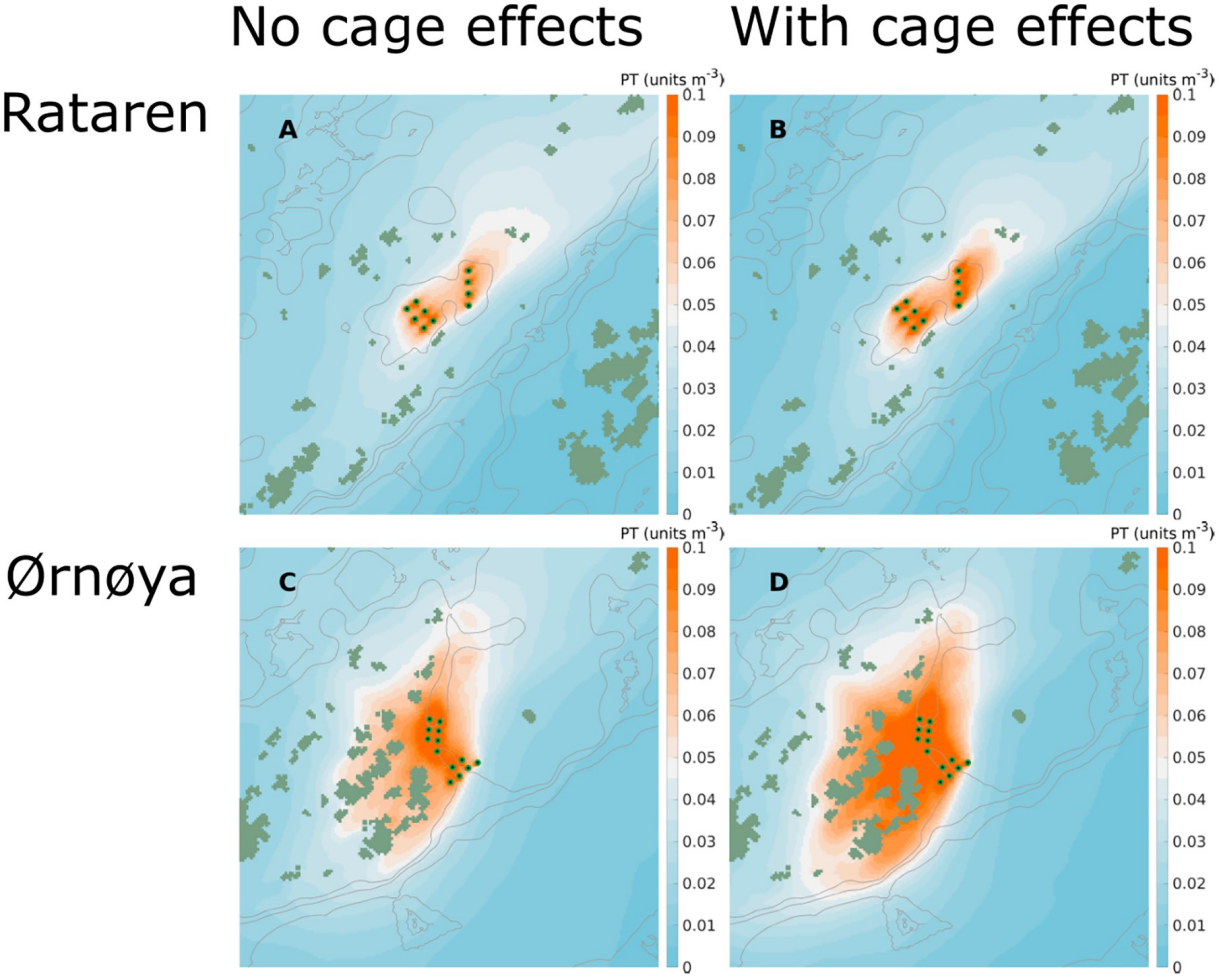
Average simulated dispersal of passive tracers in August-September, 2015. Simulated dispersal of passive tracers from the Rataren (A, B) and Ørnøya (C, D) farms with (B, D) and without (A, C) including the effects of the cages. The results are depth integrated (0 –10 m) means (24 d) of passive tracer concentrations. The gray curves in the background are 30, 50 and 100 m isobaths. The release points of the passive tracer are indicated by green-black dots.

**Fig 14:**
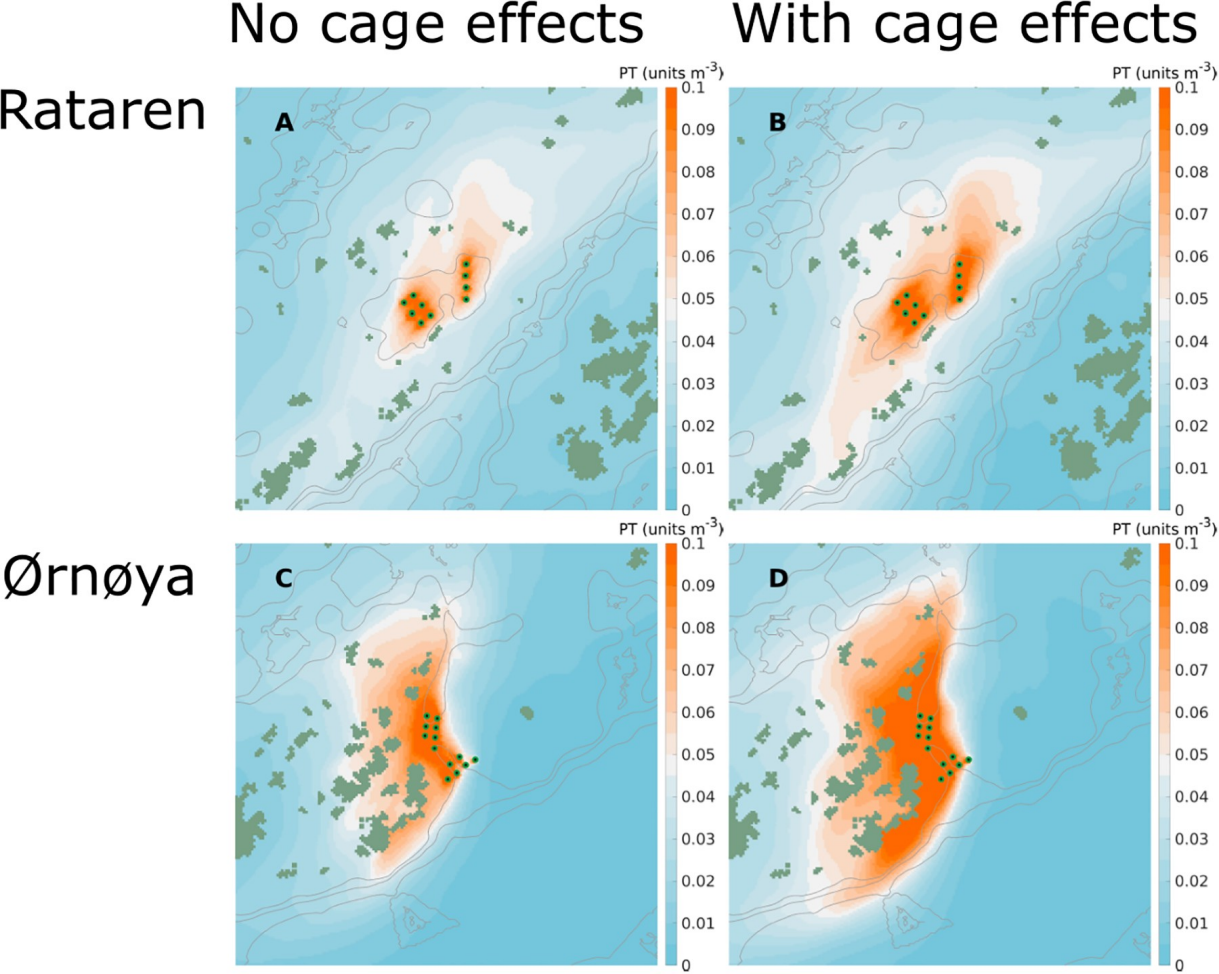
Average simulated dispersal of passive tracers in May-June, 2016. Simulated dispersal of passive tracers from the Rataren (A, B) and Ørnøya (C, D) farms with (B, D) and without (A, C) including the effects of the cages. The results are depth integrated (0 –10 m) means (24 d) of passive tracer concentrations. The gray curves in the background are 30, 50 and 100 m isobaths. The release points of the passive tracer are indicated by green-black dots.

At both farms (Fig 2) the passive tracer concentrations near the release points increased when the effect of the farm structures were taken into account. This was so both for instantaneous concentrations (Fig 12) and average concentrations over longer time periods (Figs 13 and 14).

The dispersal at the two farm sites was quite different. At Rataren, the dispersal of the PT seemed to be mainly along the SW-NE-axis (Figs 13A and 13B; 14A and 14B) both in Autumn and in Spring. This fits well with the general current pattern, both observed and simulated (Figs 7–10). At Ørnøya there was a sharp gradient in PT concentration along a section at the north western end of the farm. The PT seemed to be dispersed mainly in the western direction, at the same time being pushed somewhat around the skerries to the west.

The dispersal was greater in May than in August, at both fish farms, in particular when the effects of the cages were included (Figs 13 and 14).

The region covered by high (≥0.1 units) PT concentrations was greater at Ørnøya than at Rataren for both simulation periods. This reflects the facts that the amounts of PT released from Ørnøya were greater than at Rataren (12 vs 10 cages) and that the physical dilution was greater at Rataren (higher current speeds).

**Fig 15:**
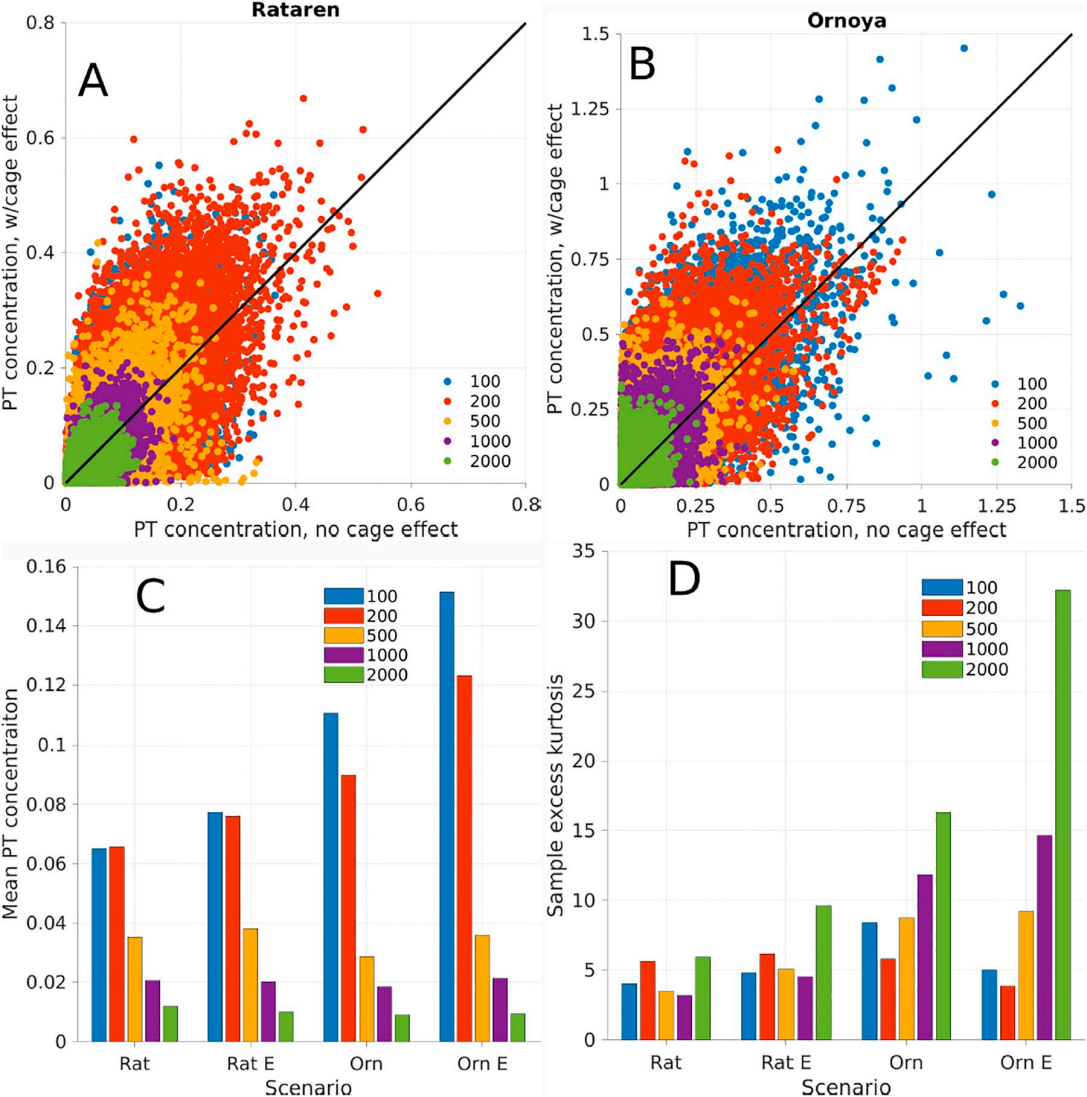
Passive tracer concentrations at selected distances from the farm centres. A, B: Scatter plots of simulated passive tracer concentrations at 10 m depth with (ordinate) and without (abscissa) cage effects at Rataren (A) and Ørnøya (B). Each point represents a sample in time and space at the distance indicated by the color from the two scenarios (Aug-Sep, 2015 and May-Jun, 2016) combined. A complete circle (excluding land points) around the farm centre was sampled with radius indicated by the colors. A 7 day spinup was used to allow the passive tracer to disperse sufficiently. C: Means of the simulated passive tracer concentrations at the distances sampled in A, B for the same time periods and for the two farms with and without cage structure effects. Inclusion of the cage strucutres is indicated by an “E” in the labelling. D: Sample excess kurtosis of the data presented in A, B, and C for each farm and scenario.

While the average dispersal patterns were different in August-Sep, 2015 and in May-June, 2016, the differences between the scenarios with and without the cage effect was greatest in May-June at both farms (compare Figs 13B and 14B). From the scatter plot of PT concentrations with cage effect against PT concentrations without cage effects in Fig 15(A) and 15(B) we see again how the cage effects increases the PT concentrations close by the farm. This is more apparent at Ørnøya than at Rataren. The PT concentrations decreased, generally speaking, with the distance from the centre of the farms, both with and without including the cage effects.

Because the same total volume of passive tracer was released in the two scenarios (with / without cage effects), higher concentrations in some points necessarily imply lower concentrations in others. This is seen at the 1000 and 2000 m samples at Rataren and at the 2000 m samples at Ørnøya (Fig 15A, 15B and 15C), where the mean concentrations are slightly lower when the cage effects are included. Despite the mean (spatiotemporal) passive tracer concentration decreasing at greater (>1000 m) distance from the farm when including the cage effects, there were more frequent instances of relatively high PT concentrations. One way to quantify this is via the *sample excess kurtosis* of the frequency distribution of PT concentrations. The sample excess kurtosis is defined for a sample *x*_1_, ⋯, *x*_*n*_ of *n* values as

$$k=\frac{\frac{1}{n}\sum_{i=1}^{n}{\left(x_{i}-\bar{x}\right)}^{4}}{{\left(\frac{1}{n}\sum_{i=1}^{n}{\left(x_{i}-\bar{x}\right)}^{2}\right)}^{2}}-3,$$

where $\bar{x}$ is the mean. Frequency distributions with positive excess kurtosis have thicker tails than the normal distribution. The excess kurtosis increased when including the farm effects, except for the values at 100 and 200 m at Ørnøya (Fig 15D). There were no great differences in the duration of periods of relatively high PT concentrations between the scenarios with and without the cage effects.

### Water contact between the farms

There were indications that the passive tracer concentrations released from either of the farms and sampled at the other (concentration sampled at each of the fish cage positions from 1 to 25 m depths in 0.5 h intervals over a period of 1 month period in August, 2015 and May, 2016) changed significantly (statistics mean and median, determined by boot strapping) by introducing the cage effects on the current field. Concentrations of the tracers released from Rataren sampled at Ørnøya were generally higher than the concentrations of the tracers released at Ørnøya sampled at Rataren.

## Discussion

This paper considers important aspects of and processes related to simulation of currents and dispersal of dissolved matter from open water aquaculture installations. Current measurements support the simulation results from the ocean model (SINMOD). In an idealized setting like a channel, the effects of the farm structures in SINMOD qualitatively match those of the CFD simulations, but in reality or in a realistic model setting are more difficult to confirm or reproduce [17]. However, the purpose of this study has not been to fully mimic or replace CFD tools, but to highlight how the inclusion of the effects of farms structures impact on the dispersal pattern of tracers in relatively realistic simulation scenarios, variables that are important in various management tools and in impact assessments.

### Simulation results and current measurements

On a qualitative level, the agreement between the simulation results for Rataren and the current measurements was good. In particular, the model reproduced the current directions well. In some cases the current speeds were too high (ADCP3 August, 2015). In other cases the spread of direction was a lot narrower in the model than the observations. At one station (ADCP1), the observations were spread out in almost all directions, while the simulations indicated currents in SW/NE direction. However, this observation point was very close to land and the bottom depth was less than 10 m, probably causing some of the differences here.

The direct correlation between simulated and observed current time series was not very good. This is quite common in ocean modelling and is the reason why studies such as this use the longer term statistical properties of the current dynamics rather than time series correlation directly to assess the skill. Despite this, ocean models can fairly accurately estimate properties such as current speed and directions. In the present study, the current roses from the observations and the model were similar, indicating that the current patterns and dispersal over time scales of days and weeks were reasonable. Except for special and critical short-term operations, it is mostly from such a time scale and upwards that simulation results of this kind are being applied in management and assessment systems.

### The drag parameter

By using a higher resolution in the selection of candidate parameters *δ* between 0.03 and 0.05 we would potentially obtain a lower RMSD in Eq (5). The effect of this would be minor, however, as seen from the slope of the RMSD-curve around *δ* = 0.04 compared with other regions (Fig 6A). Therefore, the simulation results and the conclusions derived from them are not sensitive to small perturbations of *δ*_struct_.

This conclusion is based on the simple definition of *δ*_struct_ by Eq (5) and its numerical value determined by Eq (6) that we have used throughout. More sophisticated definitions of the parameter, taking into account, e.g., the depth and the current speed and direction, would probably provide a better match between the ocean model (SINMOD) and the CFD tool (ANSYS Fluent^®^).

The use of CFD modelling for parametrization of the ocean model is a new and efficient approach. It can be seen as a way to link processes on different temporal and spatial scales. The fact remains, however, that the ocean model resolution prohibits resolving all local features in the hydrodynamics around aquaculture sea cages. Previous efforts have included parametrization based on laboratory experiments [11].

### Effects of the drag parametrization

The depth profiles of simulated currents were qualitatively similar to the results presented in [24], and show that the ocean model was able to reproduce important features including acceleration beneath the cages and reduction of currents down stream of the farm. The site in [24] has a flat bottom with a simpler current pattern. Further dissimilarities arise due to the fairly coarse resolution (32 m) of the ocean model compared with the scale of the experiment reported in [24].

The simulated depth profiles (Fig 11) also indicate that there is an effect of the cages on the currents up to at most 300 m in the present case. This explains why there was no explicit cage effect visible in the simulated nor the observed current data, in line with what is expected. Thus, the ADCP data can not be used to directly verify the effects of the cage structures on the currents.

The percentage reduction in current speed through the cage in the channel setup (Fig 4) was comparable in the Fluent (CFD) and SINMOD (ocean model) simulations, that were parametrized according to the field data in [24]. Other studies have reported different figures, e.g [29], but these are difficult to assess in the present context because they refer to either scale models or arrays of multiple cages. Previous simulation work has indicated that extensive aquaculture installations may reduce current speeds by more than 50% over large areas [9–11, 30]. These studies have focused on relatively shallow bay systems. The present results differ from these because we have focused on aquaculture facilities of limited extend in an open system.

### Implications for simulation tools and management

There were no current observations available from the Ørnøya farm, so no case comparison between observed and simulated values is possible here. The Ørnøya farm was included in the study because it demonstrates the potential for ocean modelling as an exploratory and not only prognostic tool. The Rataren and Ørnøya farms were in contrast to one another in terms of tracer concentrations, and there is also the dimension of water contact between the farms which is relevant for management systems for disease transfer.

For dispersal of organic matter in a “near field” context (up to a couple of farm diameters), the results on the current modification and tracer concentrations are both interesting. Previous work has indicated that current modification due to farm structures impacts on simulation of the potential for deposition of organic matter [12]. Because point measurements made outside a farm are not likely to comprise the full effects of the farm structures [17] (present study), this underscores the importance of high resolution model data for e.g. depositional models [3, 7]. The present CFD calculations converged at around 10, 000 iterations, which required a computer wall time of about 1 week on an 8 core cluster at 1.2 GHz. The ocean model needed 1-3 weeks to complete a 2 month simulation cycle. The ocean model covers a significantly greater geographical area, runs on a seasonal time scale, and takes into consideration atmospheric forcing and boundary conditions. The option to include biological variables and interactions [15] means that even in near “near field” contexts an ocean model may provide insights that a CFD tool cannot.

Our results are also interesting in a “far field” context. Firstly, transport models indicate a potential for “far field” dispersal of organic matter from fish farms [7] that has partially been confirmed by observations [8]. The present results indicate changes in the concentration of tracers up to several farm lengths away from the release point, and thus outside the somewhat imprecisely defined “near field” region around the farm. As a caveat, one should bear in mind that tracers interacting with other variables, or subject to degradation, half life etc, would have significantly lower concentrations in the far field [15]. Secondly, the simulation results here indicate also a statistically significant increase in the passive tracers concentrations released from one farm, sampled at another approx. 4 km (air distance) away. This indicates that farm structure effects could be of importance in model based systems for disease and pathogen transport management. In Norway, a model system on sea lice contamination simulation has been used to determine salmon aquaculture zones, and the results from this system are further used as part of the knowledge platform to determine whether the biomass in each of these zones should be allowed to increase or decrease [6]. Again, it should be noted that that it has not been proved that farm structure effects have any *practical* significance in this context.

## Conclusion

The ocean model SINMOD resolved the current fields realistically at and around the farm at Rataren. While the effect of the fish cages was not obvious from the current fields alone, by measurements or simulations, this effect manifested itself clearly in terms of passive tracer dispersal simulations. Thus, the inclusion of cage effects in aquaculture environment interactions simulations should be considered as these effects impact directly on dispersal simulations and hence the concentrations of effluents in both the near and far field zones. There were both temporal and spatial differences in how the cage structures affected passive tracer dispersal.
